# Navigation with minimal occupation volume for teleoperated snake-like surgical robots: MOVE

**DOI:** 10.3389/frobt.2023.1211876

**Published:** 2023-06-12

**Authors:** Pierre Berthet-Rayne, Guang-Zhong Yang

**Affiliations:** ^1^ The Hamlyn Centre for Robotic Surgery, Imperial College London, London, United Kingdom; ^2^ Institute of Medical Robotics, Shanghai Jiao Tong University, Shanghai, China

**Keywords:** follow the leader navigation, surgical robotics, teleoperation, redundant robots, tendon/wire mechanism, motion control, flexible robots

## Abstract

Master-Slave control is a common mode of operation for surgical robots as it ensures that surgeons are always in control and responsible for the procedure. Most teleoperated surgical systems use low degree-of-freedom (DOF) instruments, thus facilitating direct mapping of manipulator position to the instrument pose and tip location (tip-to-tip mapping). However, with the introduction of continuum and snake-like robots with much higher DOF supported by their inherent redundant architecture for navigating through curved anatomical pathways, there is a need for developing effective kinematic methods that can actuate all the joints in a controlled fashion. This paper introduces the concept of navigation with Minimal Occupation VolumE (MOVE), a teleoperation method that extends the concept of follow-the-leader navigation. It defines the path taken by the head while using all the available space surrounding the robot constrained by individual joint limits. The method was developed for the *i* ^*2*^ *Snake* robot and validated with detailed simulation and control experiments. The results validate key performance indices such as path following, body weights, path weights, fault tolerance and conservative motion. The MOVE solver can run in real-time on a standard computer at frequencies greater than 1 kHz.

## 1 Introduction

Minimally Invasive Surgery (MIS) is a well-accepted surgical technique that typically uses long, rigid instruments and an endoscope to perform surgical procedures through small incisions (Wickham, 1987; Troccaz et al., 2019). Although this technique brings many advantages for patients such as reduced access trauma, less blood loss, rapid recovery time, it, however, demands unintuitive and ergonomically difficult control of the instruments. Other issues include the loss of direct vision and depth perception and complex instrument manipulation limited by the fulcrum effect.

Recent advances in surgical robotics are aimed at overcoming these difficulties with teleoperated master-slave systems combined with articulated instruments. Most current surgical robots consist of a master console operated by the surgeon and a slave robot for relaying and executing the motion commands. Features such as motion scaling and tremor removal can be incorporated. The teleoperation method typically maps the motion of the master manipulator to the instrument tip (tip-to-tip mapping) without explicitly controlling the resulting motion of the other joints. Although this approach is sufficient for rigid instruments with a direct line-of-sight access, this is problematic for more complex flexible systems such as hyper-redundant continuum robots.

Endoscopic surgery, either via intraluminal or transluminal routes, represents an advanced MIS technique that uses natural orifices rather than body incisions to access the target lesion (Vitiello et al., 2013). This is usually done with a flexible endoscope equipped with vision and light sources, pushed and manipulated by the operator. The main advantage of this technique is that it further reduces access trauma and is able to reach to deep seated lesions. However, maintaining flexibility usually contradicts with stability and the amount of force that can be exerted. Issues related to tissue manipulation, triangulation, and stable operation within tortuous small vessels are major challenges to overcome. Furthermore, effective control of flexible endoscopes is practically difficult; issues related to looping, risk of vessel perforation, and buckling are common. Despite these limitations, highly skilled endocopists can manage to perform complex procedures such as Endoscopic Submucosal Dissection (ESD) or Peroral Endoscopic Myotomy (POEM). The performance of these procedures is highly operator dependent, often involving multi-operator working together. There is therefore a need for introducing robotics to flexible endoscopic surgery, allowing easier control and more dexterous manipulation of the endoscopes.

Recently, there have been increased interests in the development of endoscopic or single port robotic systems (Vitiello et al., 2013; Burgner-Kahrs et al., 2015). For example, the STRAS system uses a commercially available endoscope and instruments augmented with robotic actuation (Zorn et al., 2017). However, insertion and navigation are still performed manually. The MASTER system uses a standard endoscope equipped with two robotic instruments (Low et al., 2006), but the significant outer diameter of 25 mm limits its potential applications. The CYCLOPS system also uses a standard endoscope with a deployable scaffold allowing to actuate standard endoscopic instruments (Mylonas et al., 2014), but possible clinical applications are limited by the size of the scaffold. The K-FLEX developed by KAIST is a fully robotic endoscope with one passive proximal section, two active distal sections and two robotic instruments (Hwang et al., 2018), but the insertion is still performed manually. The LESS system uses a short robotic body and two manually controlled instruments (Li et al., 2017). This system was developed originally for single port applications and therefore is not applicable to natural orifice surgery. The HARP system uses two concentric structures with shape-locking capabilities, allowing the device to follow tortuous pathways (Degani et al., 2006). However, the system lacks space for inner channels which must be placed outside, thus increasing the overall outer diameter. The *iSnake* (Shang et al., 2011; Shang et al., 2012) and the latest version: the *i* ^*2*^ *Snake* (Berthet-Rayne et al., 2018a) use a short, fully active body and two robotic instruments. Therefore, this system has the potential to provide fully controlled navigation together with dexterous instruments.

With all these different architectures, one common challenge is the navigation of such robots toward the surgical site of interest (Chikhaoui and Burgner-Kahrs, 2018). Whether for performing natural orifice or single port surgery, there is a common requirement to navigate inside tortuous pathways while avoiding obstacles such as arteries or organs that could potentially injure the patient. Therefore, traditional inverse kinematics approaches such as tip-to-tip mapping are no longer applicable, and there is a need for new teleoperation and control algorithms that can handle full-body shape control of these redundant snake-like robots while being intuitive to operate.

In MIS, typical types of navigation include single port with a virtual Remote Centre of Motion (RCM) (Boctor et al., 2004; Xu et al., 2009; Cianchetti et al., 2013), motion planning of pre-determined trajectories (Alterovitz and Goldberg, 2008; Kuntz et al., 2015; Fu et al., 2018) active constraints (Kwok et al., 2009; Kwok et al., 2013), Follow-The-Leader (FTL) (Choset and Henning, 1999; Kang et al., 2016; Neumann and Burgner-Kahrs, 2016) and more general shape control (Mochiyama et al., 1999; Roesthuis and Misra, 2016). RCM is mainly aimed for single port applications with rigid instruments as only the entry point is constrained. For snake robots, this can be considered as part of the overall constraint and requiring the robot to pass through a defined point and orientation. Motion planning is a method that typically relies on pre-operative images from CT or MRI scans to determine the path to follow. This requires several steps from the segmentation of the images, to the search of a feasible path either pre-operatively or online. Finally, the registration between the patient data and the robot limits the potential use for real-time endoscopic teleoperation and inspection. When tissue motion is taken into account, dynamic active constraints can be imposed (Kwok et al., 2013). Active constraint uses geometrical models and meshes to constrain the robot in a safe zone and therefore also requires pre-operative data, registration, and is difficult to adapt to soft tissue in real-time.

During endoluminal navigation, a snake-like robot should follow the path closest to the central line to avoid exerting excessive forces on the wall of the lumen. FTL navigation can ensure the robot body will follow the path taken by the head of the device. FTL is inspired from biology, more precisely by the way snakes navigates in their environment using serpentine locomotion, where the body follows the path taken by the head (Gray, 1946). However, FTL requires that the hardware can intrinsically follow the same path, which is not always feasible. Robots with complex architecture such as the *i* ^*2*^ *Snake* (Berthet-Rayne et al., 2018a), hybrid flexible-articulated devices (Hu et al., 2019), and concentric tube robots (Dupont et al., 2010) do not have an implicit way of following the path determined by the head and require more complex control methods or task-based design to follow a desired path (Gilbert and Webster, 2013; Garriga-Casanovas and Rodriguez y Baena, 2017; Berthet-Rayne et al., 2018b). Moreover, the concept of FTL motion can be restrictive, especially if the robot is much smaller than the lumen in which it is traveling, as all the surrounding space available is not exploited. Another critical feature to have while navigating within natural, tortuous pathways is the ability to follow the exact same path while extracting the instruments. This is a critical feature which is often omitted or neglected.

**FIGURE 1:**
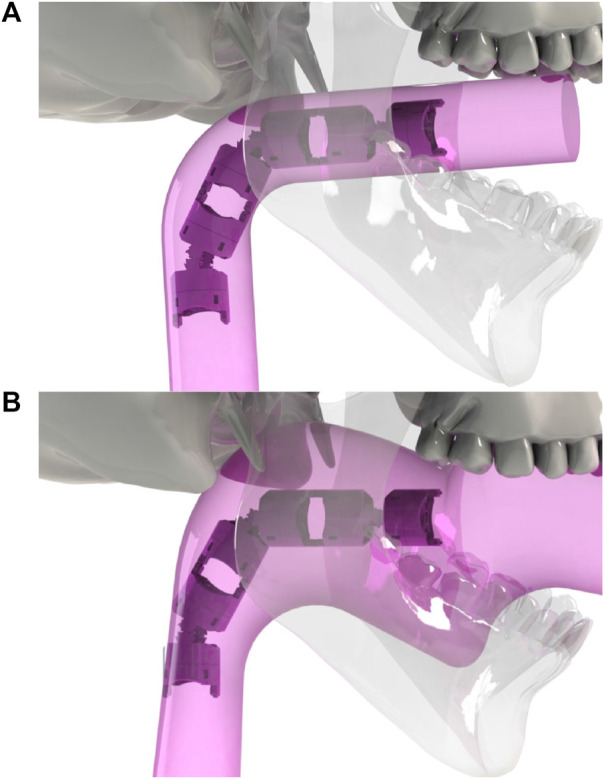
**(A)**: in a strict follow-the-leader navigation through an anatomical structure, the space that the robot occupies when reaching to the target will be equivalent to the volume of the body of the robot. **(B)**: Minimal Occupation VolumE navigation (MOVE) concept. In practice, due to joint limit and kinematic constraints, follow-the-leader is not always possible and the navigation space is larger than the volume of the robot. With MOVE, all the surrounding available space can be used.

In this paper, we introduce a novel navigation paradigm based on Minimal Occupation VolumE (MOVE). This method builds up on top of our previous work on teleoperation of highly redundant snake-like robots which highlighted the need for intuitive algorithms that would simplify the control from the user standpoint (Berthet-Rayne et al., 2018b). The basic concept of MOVE is illustrated in Figure 1. It is a generic method that can be used for different flexible robot designs or procedures. This includes endoscopic surgery (colonoscopy, gastroscopy), bronchoscopy, catheterisation, or non-medical applications such as pipe and jet engine inspection as well as search and rescue exploration. This navigation concept extends the FTL method in the sense that it uses the path taken by the head as a guide rather than a strict path to follow, giving some freedom for optimized use of all the available space surrounding the robot. The main difference and advantage of the MOVE navigation is that it allows robots with complex architecture to reach deep-seated lesions while navigating through curved lumens as all the available space around the robot can be used to compensate for the robot’s kinematic limitations. This extra space can also be used to enhance the dexterity of the robot or reduce mechanical stress, which could help to perform safer and more complex surgeries. Finally, MOVE navigation can ensure safe retraction of the robot by following the exact same path as the one used to navigate inside.

This paper is structured as follow: Section 2 introduces the concept and method of MOVE in details. Section 3 presents the validation and results on the *i* ^*2*^ *Snake* simulator. Section 4 outlines the implementation on the real *i* ^*2*^ *Snake* robot. This is followed by a discussion in Section 5 and the conclusion and future work in Section VI.

## 2 Methods

The MOVE navigation combines teleoperation and navigation of redundant snake-like robots. The motivation is to allow snake-like robots with non-holonomic joints, *i.e.*, joints that can only move on a single plane, to follow an unknown-in-advance 3D path in serpentine-like locomotion by allowing the use of available surrounding space. The method relies on differential inverse kinematics with the capability to perform full-body shape control. The approach consists of four key phases: {navigation, path creation, virtual robot fitting, inverse kinematic}.

**FIGURE 2:**
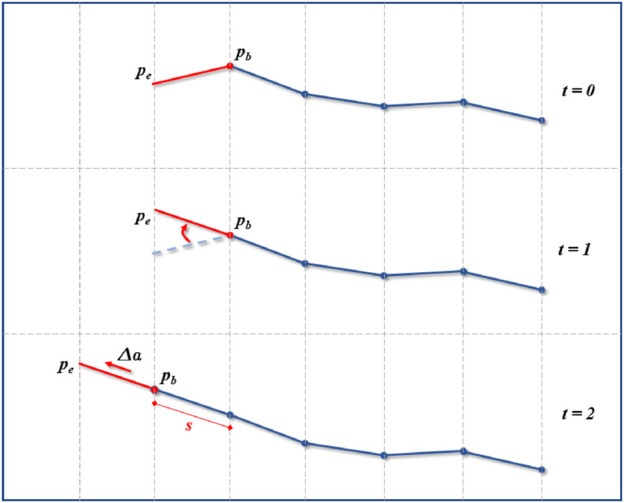
Navigation and path creation. The operator can control the head (red segment) orientation by moving $\vec{p_{e}}$ around $\vec{p_{b}}$ . During the insertion motion Δ*a*, the base of the head $\vec{p_{b}}$ will determine the new path points (in red) at a fixed sampling distance *s*.

These can be summarized as follows. The head of the robot is controlled by the surgeon in a similar fashion as in traditional endoscopy. The operator can control the roll, pitch, yaw and forward/backward motion of the robot. The forward motion is determined by the head orientation as depicted in Figure 2. As the head is moving forward, the path taken by the base of the head is sampled with a fixed resolution. As the path grows, a virtual robot with the same kinematic structure (same amount of link and link length) than the real one, but with universal joints (3 DOFs) between each links instead, is fitted to the path by using back-propagation from the head to the base. This virtual robot is then used to generate target points along the path to solve the full-body inverse kinematics of the real robot. This allows the surgeon to intuitively navigate in real-time along the desired path without worrying about the complexity of the robot’s kinematic structure. Each step and its corresponding equations are further described below.

### 2.1 *i* ^*2*^ *Snake* kinematics

To illustrate how the proposed method works in practice, we used the *i* ^*2*^ *Snake* robot as an example to demonstrate the functionalities of the algorithm. As MOVE uses the Denavit-Hartenberg (DH) convention, this framework would also work on other types of articulated robots.

The detailed forward kinematics of the *i* ^*2*^ *Snake* was previously presented in (33). The *i* ^*2*^ *Snake* is a tendon-driven articulated robot composed of 12 rolling-joints arranged orthogonally. The 12 joints are grouped into 3 sections named proximal, middle and distal and having 4 DOFs each, which are then mechanically coupled into 2 DOFs. The base of the *i* ^*2*^ *Snake* further provides 1 DOF roll motion. The *i* ^*2*^ *Snake* is then attached to a robotic arm providing the insertion motion required to navigate inside the patient. All together, the *i* ^*2*^ *Snake* presented in (33) has 8 controllable DOFs. However, due to the type of joint used (rolling-joint) and its particular rolling motion, the *i* ^*2*^ *Snake* DH table requires 26 joint variables with intrinsic mechanical coupling. The reader can refer to (Berthet-Rayne et al., 2018b; Berthet-Rayne et al., 2018c; Berthet-Rayne et al., 2019) for further details on the *i* ^*2*^ *Snake* design, kinematics and the joint coupling.

In the context of this paper, the *i* ^*2*^ *Snake* kinematics is further augmented with additional 4 DOFs to allow 6 DOFs operation at the base of the robot and fully exploit the robotic arm holder capabilities as shown in Table 1, lines 1 to 6.

**TABLE 1:** Extended DH table of the *i* ^*2*^ *Snake* robot.

***i***	a	***α***	***d***	***θ***	Type	Coupling
1	0	*π*/2	*d*_1_	*π*/2	P	0
2	0	*π*/2	*d*_2_	*π*/2	P	1
3	0	*π*/2	*d*_3_	0	P	2
4	0	*π*/2	0	*θ*_1_	R	3
5	0	*π*/2	0	*θ*_2_	R	4
6	0	*π*/2	*a*_0_	*θ*_3_	R	5
7	0	*π*/2	0	*θ*_4_	R	6
8	*a*_1_	0	0	*θ*_5_	R	6
9	*a*_2_	*π*/2	0	*θ*_6_	R	7
10	*a*_1_	0	0	*θ*_7_	R	7
11	*a*_2_	-*π*/2	0	*θ*_8_	R	6
12	*a*_1_	0	0	*θ*_9_	R	6
13	*a*_2_	*π*/2	0	*θ*_10_	R	7
14	*a*_1_	0	0	*θ*_11_	R	7
15	*a*_2_	-*π*/2	0	*θ*_12_	R	8
16	*a*_1_	0	0	*θ*_13_	R	8
17	*a*_2_	*π*/2	0	*θ*_14_	R	9
18	*a*_1_	0	0	*θ*_15_	R	9
19	*a*_2_	-*π*/2	0	*θ*_16_	R	8
20	*a*_1_	0	0	*θ*_17_	R	8
21	*a*_2_	*π*/2	0	*θ*_18_	R	9
22	*a*_1_	0	0	*θ*_19_	R	9
23	*a*_2_	-*π*/2	0	*θ*_20_	R	10
24	*a*_1_	0	0	*θ*_21_	R	10
25	*a*_2_	*π*/2	0	*θ*_22_	R	11
26	*a*_1_	0	0	*θ*_23_	R	11
27	*a*_2_	-*π*/2	0	*θ*_24_	R	10
28	*a*_1_	0	0	*θ*_25_	R	10
29	*a*_2_	*π*/2	0	*θ*_26_	R	11
30	*a*_1_	0	0	*θ*_27_	R	11

### 2.2 Navigation

During endoscopic surgery, the tip of the endoscope is usually equipped with an imaging system such as a camera or an optic fibre bundle. In traditional endoscopy, 4 DOFs are available to the endoscopist: the roll, the pitch, the yaw, and the insertion, all controlled manually from the back of the endoscope and using the video. Therefore, the same 4 DOFs are also used on the proposed robotic approach. The head of the robot, where the camera is installed, is determined by two 3D points, one at the base $\vec{p_{b}}$ and one at the tip $\vec{p_{e}}$ forming a vector $\vec{v_{\mathrm{head}}}=\vec{p_{e}}-\vec{p_{b}}$ . The orientation of the head is controlled by rotating $\vec{v_{\mathrm{head}}}$ with the rotation matrix *R*_*head*_ (containing the roll, pitch and yaw angles) around the point $\vec{p_{b}}$ as follow:

$$\vec{p_{e}}=R_{\mathrm{head}}\;\vec{v_{\mathrm{head}}}+\vec{p_{b}}$$ (1)

The insertion motion Δ*a* will induce a translation of *v*_*head*_ along itself:

$${\left[\vec{v_{\mathrm{head}}}\right]}_{t+1}=\Delta a\;{\left[{\hat{v}}_{\mathrm{head}}\right]}_{t}$$ (2)

where ${\left[{\hat{v}}_{\mathrm{head}}\right]}_{t}$ represents the normalized vector $\vec{v_{\mathrm{head}}}$ at time *t*, and ${\left[\vec{v_{\mathrm{head}}}\right]}_{t+1}$ the translated vector $\vec{v_{\mathrm{head}}}$ at time *t* + 1. This process is represented geometrically in Figure 2. Once the position and orientation of the head known, the next step is the path creation.

### 2.3 Path creation

The head, more specifically the base of the robot head $\vec{p_{b}}$ , is used to determine the path to be followed by the rest of the body. Every time the insertion is changed by the operator, the vector $\vec{v_{\mathrm{ins}}}$ between ${\left[\vec{p_{b}}\right]}_{t}$ and ${\left[\vec{p_{b}}\right]}_{t+1}$ is computed and its corresponding norm *d* is calculated. If *d* is equal or greater than the sampling resolution *s*, a new point is added to the *path* at index *m*. This process is depicted in Figure 2 and summarized in the Algorithm 1.

Algorithm 1 Path Creation

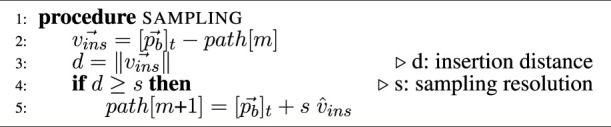

Only points that correspond to a change in insertion are saved as the operator might explore the surroundings by changing the head orientation before moving forward toward the site of interest.

### 2.4 Virtual robot fitting

A virtual robot is used to identify target points for the real robot along the path. This virtual robot is a modified version of the real robot being controlled. It has the same amount of links and same link length. The only difference is that each revolute joint is modelled as a universal joint with 3 DOFs. This feature ensures that the virtual robot’s link ends can be fitted exactly on the desired path as in typical FTL navigation.

The head link of the virtual robot is known from the previous navigation step. The rest of the link’s position is determined using a backward fitting (from head to base) onto the path. In this section, the first link will refer to the head and the following links will be starting from the head, going towards the base of the robot. The fitting consists of mapping each extremity of each link onto the path. This is done in an iterative process for the *n* DOFs, with each link’s end being the start of the next one, and is repeated until the base is reached. The head link extremities are already known, so the next step is to fit the remaining link’s end onto the path. Two cases can arise while doing so.

#### 2.4.1 Fitting case 1

The first one is when the distance *d* between the link’s end $\vec{p_{e}}$ and the current path point *path* [*j*] at index *j* is longer than the link length *l*. In this case, it is required to interpolate between the link’s end and the path point, and to find the intersection coordinates *x* between a sphere of radius *r* = *l* and the line formed by the previous path point *path* [*j* − 1] and current path point *path* [*j*] using the following equations (Glassner, 1989):

$$\vec{x}=\vec{A}+\left(-\hat{u}\;•\;\left(\vec{A}-\vec{p_{e}}\right)+\sqrt{\Delta }\right)\;\hat{u}$$ (3)

with

$$\Delta ={\left(\hat{u}\;•\;\left(\vec{A}-\vec{p_{e}}\right)\right)}^{2}-\|\vec{A}-\vec{p_{e}}{\|}^{2}+r^{2}$$ (4)

and

$$\hat{u}=\frac{\vec{B}-\vec{A}}{\left|\vec{B}-\vec{A}\right|}$$ (5)

where $\vec{A}$ is the previous path point *path* [*j* − 1], $\vec{B}$ is the current path point *path* [*j*], and • represents the dot product.

#### 2.4.2 Fitting case 2

**FIGURE 3:**
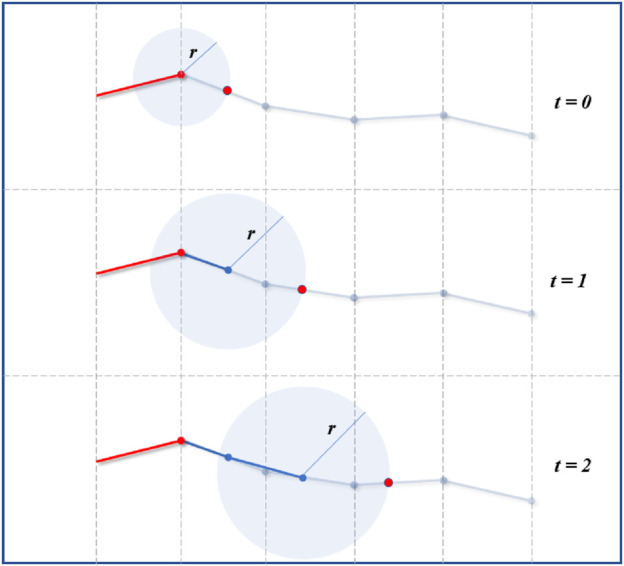
Virtual robot fitting by back-propagation onto the path. Sphere line intersection is used to find where each link with different lengths fits onto the path.

The second case is when the distance *d* between the link’s end $\vec{p_{e}}$ and the current path point *path* [*j*] at index *j* is shorter than the link length *l*. In this case, the path index must be decremented until the fitting case 1 is valid. Then, the point on the path corresponds to the intersection between the sphere of radius *r* = *l* and the line formed by the previous and current path point, similarly as in case 1. This algorithm is represented in Figure 3 and is summarized in the Algorithm 2.

Algorithm 2 Virtual Robot Fitting

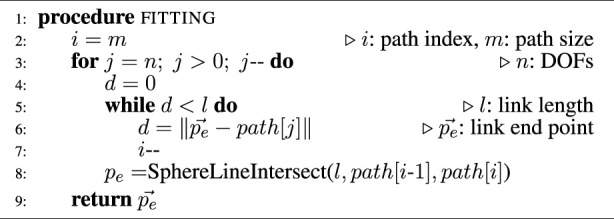

### 2.5 Inverse kinematics

Now that all the intermediary target points are known, the next step consists of finding an inverse kinematics solution for the robot to follow the desired path. Conventional inverse kinematics algorithms consider only the end effector of the robot. The Jacobian approach allows to iteratively find a solution to place the tip of the robot in a desired position and orientation without explicitly considering intermediary joints. The Jacobian is defined as follow (Siciliano et al., 2010):

$$v_{e}=\left[\begin{array}{c} {\dot{p}}_{e}\\ \omega_{e} \end{array}\right]=J\left(q\right)\;\dot{q}$$ (6)

where *v*_*e*_ is the end effector velocity, *J* (6 × *n*) is the geometric Jacobian matrix of the robot defined in the base frame, *n* is the robot’s DOFs, ${\dot{p}}_{e}$ is the linear velocity of the tip, and *ω*_*e*_ the angular velocity with *J* defined as follow (Siciliano et al., 2010):

$$J=\left[\begin{array}{ccc} J_{P_{1}} & & J_{P_{n}}\\ & \ldots \\ J_{O_{1}} & & J_{O_{n}} \end{array}\right]$$ (7)

where $J_{P_{i}}\;\left(3\times 1\right)$ and $J_{O_{i}}\;\left(3\times 1\right)$ relate to linear velocities and angular velocities respectively. $J_{P_{i}}$ is calculated iteratively as follow (Siciliano et al., 2010):

$$J_{P_{i}}=\left\{\begin{array}{cc} z_{i-1}\; & \;\text{for a prismatic joint}\\ z_{i-1}\times \left(p_{e}-p_{i-1}\right)\; & \;\text{for a revolute joint} \end{array}\right.$$ (8)

and $J_{O_{i}}$ is calculated iteratively as follow (Siciliano et al., 2010):

$$J_{O_{i}}=\left\{\begin{array}{cc} 0\; & \;\text{for a prismatic joint}\\ z_{i-1}\; & \;\text{for a revolute joint} \end{array}\right.$$ (9)

In order to follow a desired path in a serpentine motion, one must consider all the joints of the kinematic chain. To this end, we introduce herewith the concept of a full-body Jacobian matrix *J*_*F*_. The concept of extending/augmenting the Jacobian of a manipulator to consider additional constraints such as joint limits or obstacles is known in the literature (Sciavicco and Siciliano, 1988; Slotine, 1991). In the context of this paper, the proposed full-body Jacobian is combined with a full-body inverse kinematic algorithm allowing to control all the joints individually and in a controlled way.The objective of the proposed algorithm is to minimize the error between each path point and each link’s end of the robot. As the path points are defined in 3D space, only the Cartesian distance is needed for the intermediate body joints, so only the linear velocity need to be considered. The head of the robot however needs to be controlled in orientation as well, hence only the angular velocity of the robot’s tip need to be computed. As a result, the extended Jacobian *J*_*F*_ ((3*n* + 3) × *n*) stacks the Jacobians of each link as if it was an end effector and is defined as follow.

$$J_{F}=\left[\begin{array}{ccc} J_{P_{11}} & & J_{P_{1n}}\\ J_{P_{21}} & & J_{P_{2n}}\\ & \ldots \\ J_{P_{n1}} & & J_{P_{nn}}\\ J_{O_{1}} & & J_{O_{n}} \end{array}\right]$$ (10)

where $J_{P_{ii}}\;\left(3\times n\right)$ and $J_{O_{i}}\;\left(3\times n\right)$ relates to linear velocities of each link’s end of the robot calculated as a traditional Jacobian (Siciliano et al., 2010). Entries of the Jacobian which are not defined (first columns of first links) are set to 0 as shown in the Algorithm 3.

Algorithm 3 full-body Jacobian Computation

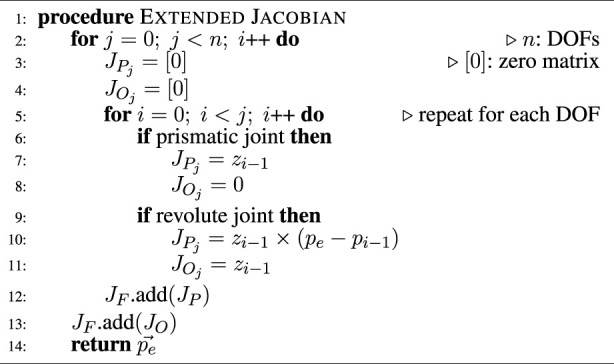

Finally, the full-body inverse kinematics equation can be expressed as follow:

$$\dot{q}=J_{F}^{†}\left(q\right)v_{f}$$ (11)

where $J_{F}^{†}\left(q\right)$ is the Moore-Penrose pseudo-inverse of *J*_*F*_ computed using a singular value decomposition, and *v*_*f*_ is the full-body velocity vector. Eq. 11 can be used to solve for the full-body inverse kinematics by integrating the velocities over time iteratively (Buss, 2009):

$$q_{t+1}=q_{t}+\alpha \;J_{F}^{†}\left(q_{t}\right)\;e_{f}$$ (12)

with *α* a scalar that can be used to scale the convergence at each iteration, and *e*_*f*_ ((3*n* + 3) × 1) is the full-body error between the current robot position and the desired path target points, defined as follow:

$$e_{f}=\left[\begin{array}{c} e_{P}\\ e_{O} \end{array}\right]$$ (13)

with *e*_*P*_ (3 *n* × 1) the position error and *e*_*O*_ (3 × 1) the orientation error of the snake’s head. *e*_*P*_ is calculated as follow:

$$e_{P}=p_{d}-p_{e}\left(q\right)$$ (14)

with *p*_*d*_ (3 *n* × 1) the vector of desired target points, and *p*_*e*_ (3 *n* × 1) the vector of the current state of each link. *e*_*O*_ is computed using the angle and axis approach (Siciliano et al., 2010):

$$e_{O}=\beta \;sin\left(\vartheta \right)$$ (15)

with *β* and *ϑ* representing the angle and axis respectively of the rotation between the current robot orientation and the desired orientation. Eq. 12 is the main equation used to find a local optimal solution that will bring the joints as close as possible to the desired path. This iterative equation is used every time the head of the robot is moved. Therefore, using (12) in real-time would result in a FTL-like motion, where the robot follows the path in the best physically possible way.

However, the concept of MOVE consists of using all the available surrounding space which Eq. 12 cannot fulfill. To allow this extended feature, this paper proposes to add a weighting matrix to the iterative Eq 12. The goal of the weighting matrix is to assign a weight to each link’s end which will determine the degree of importance to bring this specific link close to the path. Higher weights mean that the point must be as close as possible to the path, while lower weights will result in the link’s end being further away. As the robot moves inside the lumen, the weight can be altered *in-situ* to obtain different behaviours depending on the available space around the links of the robot. In the latter scenario, the weights can be set from various types of sensors, such as tactile, stereo-vision, infrared, ultrasonic, *etc*. The final weighted iterative equation is shown below:

$$q_{t+1}=q_{t}+\alpha \;J_{F}^{†}\left(q_{t}\right)\;W\left(e_{f}\right)\;|e_{f}|$$ (16)

with *W* (*e*_*f*_) ((3 *n* + 3) × (3 *n* + 3)) the diagonal weighting matrix calculated using the error vector. There is a vast variety of weighting scenario that can be used to calculate *W* (*e*_*f*_) depending on the final application, the sensing method used and whether the signal is discrete or continuous.

**FIGURE 4:**
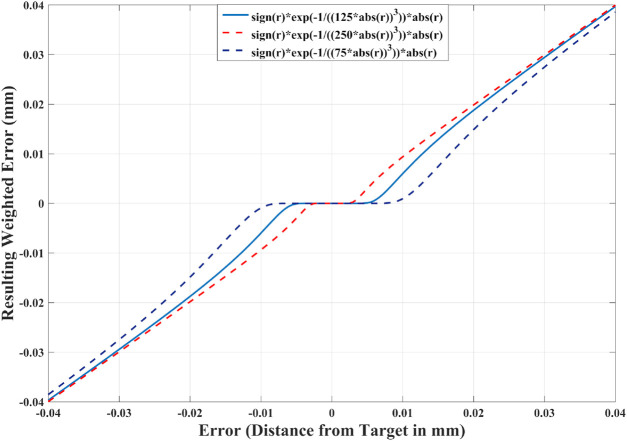
Weighting function used for MOVE. When the error is large, it is not affected as the function is close to be linear. As the error decreases, it is continuously attenuated by a pre-determined threshold. Three different examples of attenuation are plotted here.

One possible approach is to allow each link of the robot to be within a set distance of the target. If the robot is further from this threshold, the robot should converge toward the solution; as the link approach, they should continuously slow down the convergence until the desired threshold is reached. This can be done using the following equation to calculate *W* (*e*_*f*_):

$$W_{ii}=\left\{\begin{array}{cc} sign\left(e_{f_{i}}\right)\left(1-w_{i}\right)e^{-\frac{a}{{\left(b|e_{f_{i}}|\right)}^{3}}}\; & \;\text{if }e_{f_{i}}\neq 0\\ 0\; & \;\text{if }e_{f_{i}}=0 \end{array}\right.$$ (17)

with *w*_*i*_ a scalar allowing to turn weighting on (*w*_*i*_ = 1) or off (*w*_*i*_ = 0), *a* and *b* two scalars than can be used to set the desired threshold distance. An example of used values is plotted in Figure 4. The advantage of using Eq. 17 is that the error is almost unaffected while outside the threshold distance as the function will tend to 1 and results in a linear function, and it will quickly and continuously attenuate the error once within the desired threshold distance and as a result stop the convergence of Eq 16. As some joints will be allowed to move more as they are less important to the solution, it is then possible to exploit the null-space of the robot to add a second constraint to the optimization (Liegeois, 1977) such as staying as close as possible to the joint centre position to reduce tendon stress due to excessive joint bending and resulting friction. This can be done as follow:

$$q_{t+1}=q_{t}+\alpha \;J_{F}^{†}\left(q_{t}\right)\;W\left(e_{f}\right)\;|e_{f}|+\left(I-J^{†}\left(q_{t}\right)J\left(q_{t}\right)\right)\varphi$$ (18)

with *φ* the secondary objective function allowing to reduce the tendon stress by keeping the joints as close as possible to their centre position, and is defined as follow (Girard and Maciejewski, 1985):

$$\varphi =\nabla H\text{ and }H=\overset{n}{\underset{i=1}{\sum }}\eta_{i}{\left(\theta_{i}-\theta_{c_{i}}\right)}^{2}$$ (19)

with *η*_*i*_ a scalar acting as a gain with values between [0,1], and $\theta_{c_{i}}$ the centre value of joint *i*. It is important to note that Eq. 18 uses both the full-body Jacobian and the standard Jacobian as defined in Eq. 7.

The final feature of MOVE is to allow conservative retraction motion by using the same path than the one used during the insertion and is discussed in the next Section.

### 2.6 Conservative motion

Conservative motion is a critical feature in endoscopic surgery. As the robot navigates inside the lumen, the surgeon uses the vision feedback combined with his/her situation awareness to find the safest path to follow. Therefore, the robot must follow the exact same path during the retraction phase which cannot be insured with traditional tip control. As the path is sampled and saved during the path creation phase, the proposed navigation framework has the intrinsic property of being conservative.

### 2.7 Mechanical fault tolerance

The proposed navigation approach, using all the available space, and trying to find the best fit to the path, can also be exploited to compensate for potential mechanical failures that can arise during surgery. Tendons are ideal for miniaturization and remote force transmission, but are also prone to failures if exposed to excessive forces or friction. Types of failure include, for example,: complete snapping, broken core, bird-caging, kinking, and it is safer to stop the actuation when any type of failure occurs. Typical tendon driven instruments on existing surgical robots are therefore restricted to a limited amount of use to avoid the risk of such scenarios from happening, but such failures are still known to occur (Freschi et al., 2013). Although rigid instruments can often be extracted and replaced quickly, in the case of flexible systems, the loss of one or multiple tendons could potentially prevent the safe extraction of the robot. In the case of the *i* ^*2*^ *Snake* robot, there is a considerable amount of tendons used as each pair of joint is connected to 2 tendons (26 tendons). Considering that a tendon failure can be detected by either monitoring the motor current, or by measuring the tendon tension with sensors, the proposed MOVE framework has the intrinsic capability to compensate for the loss of one or more DOFs. In (33), we introduced a method to handle joint-limits by modifying the corresponding Jacobian columns. This approach can be further extended to also handle mechanical failures and ensure a fail-safe mode allowing the operator to finish the procedure and safely extract the robot. The full-body Jacobian *J*_*F*_((3*n* + 3) × *n*) presented earlier can be represented as an aggregate of column vectors (Ben-Gharbia et al., 2014):

$$J_{F}=\left[\begin{array}{cccc} j_{1} & j_{2} & \ldots & j_{n} \end{array}\right]$$ (20)

Where each column *j*_*i*_ represents the contribution of joint *i*’s velocity toward the movement of the robot in Cartesian space. In the case where a tendon attached to joint *i* is found to be faulty, the corresponding column *j*_*i*_ will be set to the column vector zero as follow:

$$j_{i}=\left\{\begin{array}{cc} \vec{0}\; & \text{if tendon }i\text{ is faulty}\\ j_{i}\; & \text{otherwise} \end{array}\right.$$ (21)

Using Eq. 21 reflects the loss of one or multiple DOFs in the least square equations. As the column is set to zero, the contribution of that joint is voided and the corresponding joint value *θ*_*i*_ will not be changed anymore. As the MOVE framework tries to minimize the distance from the path while allowing some free space around it, Eq. 11 now considers the lost DOF and finds the best fit to the desired path. As the robot is redundant, the remaining operational joints will therefore move to compensate for the faulty one(s).

## 3 Implementation and results

As the implementation of the MOVE requires specific features such as the full-body Jacobian and real-time performance, a custom C++ library was developed: EndoRob (Berthet-Rayne, 2018). EndoRob is an open-source, cross-platform, multi-threaded robotics library allowing to do forward kinematics, iterative inverse kinematics (Jacobian transpose, pseudo-inverse, damped least square, null-space, full-body *etc.*) and can be found here: http://takskal.free.fr/EndoRob/. The code depends only on the standard c++ library as well as Eigen for the linear algebra (Guennebaud and Jacob, 2010). The algorithm was running on a standard Desktop computer with an i7-4790 CPU (Intel, USA) and 16 GB of RAM. The simulator presented in (33) was used to teleoperate the *i* ^*2*^ *Snake* in a clinically relevant environment.

### 3.1 Basic solver

**FIGURE 5:**
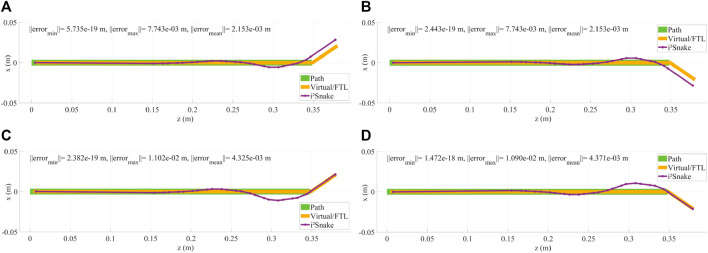
**(A,B)** Examples of head control using a basic solver. As the *i* ^*2*^ *Snake* joints are mechanically coupled and are not omni-directional, the robot cannot exactly follow the path, resulting in control inaccuracies. **(C,D)** Example of head control with a high weight on the head and on the base of the *i* ^*2*^ *Snake*. The resulting fitting is more accurate on the head position than without any weight.

**FIGURE 6:**
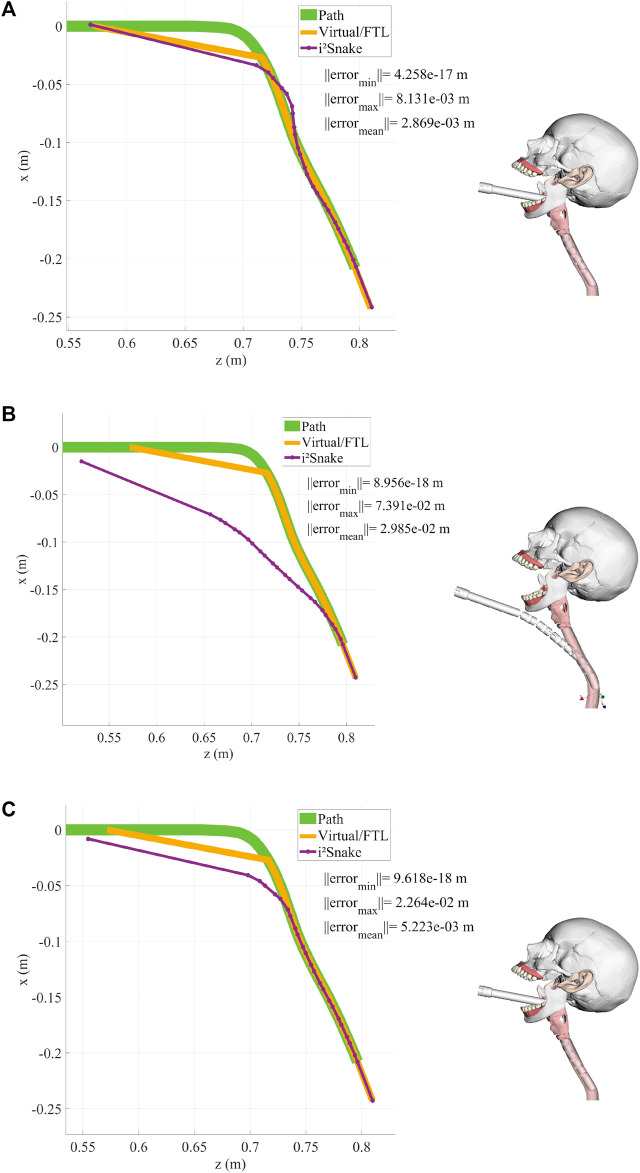
This figures shows the *i* ^*2*^ *Snake* during an insertion motion with the corresponding head path, virtual fitting (FTL motion) and robot fitting. **(A)** Basic solver without any navigation weight. **(B)** High weight on the head and a large error tolerance. **(C)** High weight on the head and a small error tolerance.

The results of the implementation of Eq. 12, which is a basic full-body inverse kinematics solver, are shown in Figures 5A, 5B, 6A. In this case, the *i* ^*2*^ *Snake* is fitted to the path without weights or tendon-stress reduction. It can be seen in Figures 5A, B that the virtual fitting (pure FTL navigation) matches the head’s path perfectly and that the robot’s link are fitted as close as mechanically feasible to the path to minimize the overall error norm.

As the robot’s joints are mechanically constrained, and therefore are not free to move in all directions, it can be seen that several joints need to be actuated to match the desired *i* ^*2*^ *Snake*’s head position and orientation. As a result, there is a significant difference between the expected and real head position, which is not a desired behaviour for surgical teleoperation as the robot’s head should be exactly where the surgeon specified during teleoperation.

**FIGURE 7:**
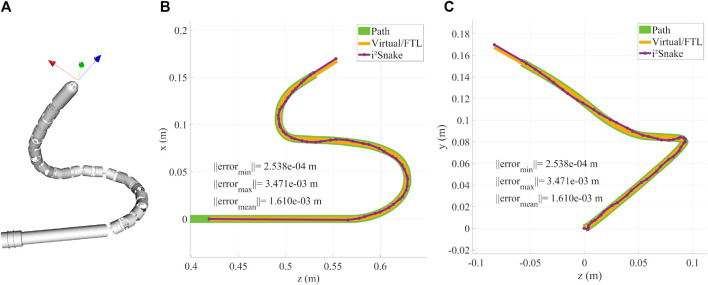
**(A)** Rendering of the *i* ^*2*^ *Snake* following a 3D coil like trajectory. **(B)** XZ plane projective view and characterization. **(C)** YZ plane projective view and characterization.

The basic solver still allows to follow complex 3D motion as depicted in Figure 7 where the *i* ^*2*^ *Snake* is fitted onto a coil-like trajectory with limited error.

### 3.2 Navigating toward the throat

Although the basic solver does not guarantee that the robot’s head is exactly on target, its performance was also evaluated during a simulated navigation down the throat as shown in Figure 6A. It can be seen that the robot is able to navigate down the oesophagus in a FTL-like fashion. This basic-solver results show the potential of the MOVE framework as the features introduced inSection 2 (body weights, error tolerance, path weights, stress reduction, and mechanical fault tolerance) will be added.

### 3.3 Weighted solver

The results of the weighted solver presented in Eq. 16 are shown in Figures 5C, D. In this example, the head and the base of the *i* ^*2*^ *Snake* were given the highest weight while all the other joints were assigned the lowest weight. This results in the control of the head being more accurate than without any weights. This can be seen in Figures 5C, D as the head follows the desired path exactly at the cost of the other joints moving to compensate for the desired configuration. This behaviour is still unsafe for clinical use as the joint motion of low-weighted joints is still not controlled and can result in undesired configuration.

### 3.4 Weighted solver with error tolerance

Introducing a controlled error tolerance is key to safe navigation inside a lumen. This can be done by implementing Eq. 16 into the solver. The results with different error tolerances are shown in Figure 6B (large tolerance) and in Figure 6C (small tolerance). In this case, the error tolerance is manually assigned to the individual *i* ^*2*^ *Snake*’s joints, but in practice this information would be sensor dependent and would reflect the surrounding anatomy’s shape.

### 3.5 Extended *i* ^*2*^ *Snake*

**FIGURE 8:**
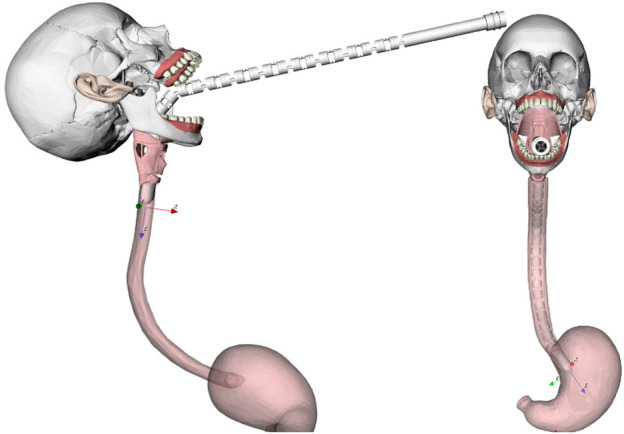
Example of navigation of the extended *i* ^*2*^ *Snake* going through the mouth. The user only controls the head, and the rest of the body will follow the head’s path as much as mechanically feasible.

In order to further demonstrate the key capabilities of the MOVE framework, in the rest of this paper, the *i* ^*2*^ *Snake* robot was extended by doubling its length and amount of joints. This can be done by adding the entries 7 to 30 at the end of the DH Table 1. This results in a new hyper-redundant extended *i* ^*2*^ *Snake* robot with 54 joint variables as shown in Figure 8. The longer *i* ^*2*^ *Snake* robot can reach further down to the stomach. Figure 8 shows an example of insertion.

### 3.6 Path weights

**FIGURE 9:**
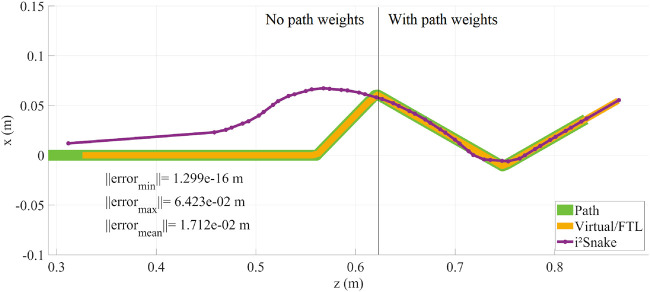
Path weights implementation result. As the *i* ^*2*^ *Snake* is navigating, the path weights are dynamically assigned to the robot’s joints. In this figure, the body of the *i* ^*2*^ *Snake* was divided in two parts. On the left: no path weights were assigned, and on the right: with path weights assigned.

The concept of weights can be further extended so the weights are not assigned to the robot directly but rather to the path itself. As the *i* ^*2*^ *Snake* is navigating and the path is created, path weights can be assigned to the path points using sensor information. As the robot navigates, these path weights can be dynamically assigned to the joints passing the corresponding path points. This method has the advantage that it creates a map of large and narrow passageway and can be integrated with sensing modalities such a stereovision or tactile sensing. The result is shown in Figure 9, where both high path weights (right side) and no path weights (left side) are assigned to two-halves of the *i* ^*2*^ *Snake*’s body during navigation. The advantage of using sensor information is that the weights would be continuous based on the sensor data and would result in smoother trajectory profiles.

### 3.7 Tendon stress reduction

**FIGURE 10:**
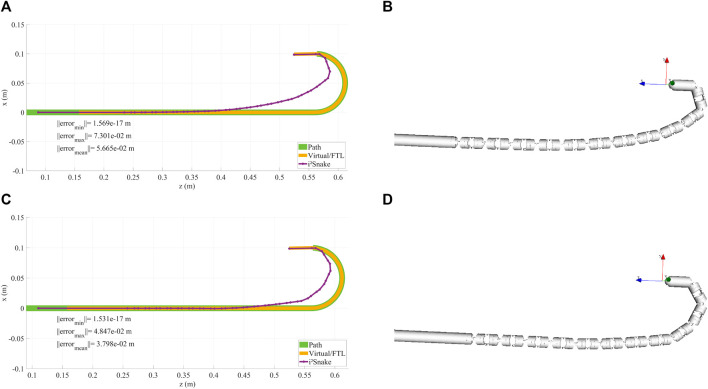
**(A,B)** Retroflex motion without tendon stress reduction. **(C,D)** same motion with stress reduction. MOVE can use the available surrounding space to avoid sharp bending joint angles. For the same trajectory, it can be seen that more joints are moving but to a smaller amount. As configuration **(A)** reached joint limit, configuration **(C)** can still move further.

The results of the tendon stress reduction are depicted in Figure 10. The tendon stress reduction was evaluated during a retroflex motion with a large error tolerance. Having a large error tolerance allows for better tendon stress reduction as more space can be used to move the intermediary joints. Figures 10A, B have no tendon stress reduction, resulting in some joints having sharp bending angles. Figures 10C, D show the exact same trajectory but with tendon stress reduction. The resulting robot configuration spreads the joints angle more evenly and has less sharp bending angles. A rendering of the robot in the same two configuration is shown in Figure 10 which shows that for the same trajectory, configuration a) reached joint limit, while configuration c) can still move further.

### 3.8 Fault tolerance

**FIGURE 11:**
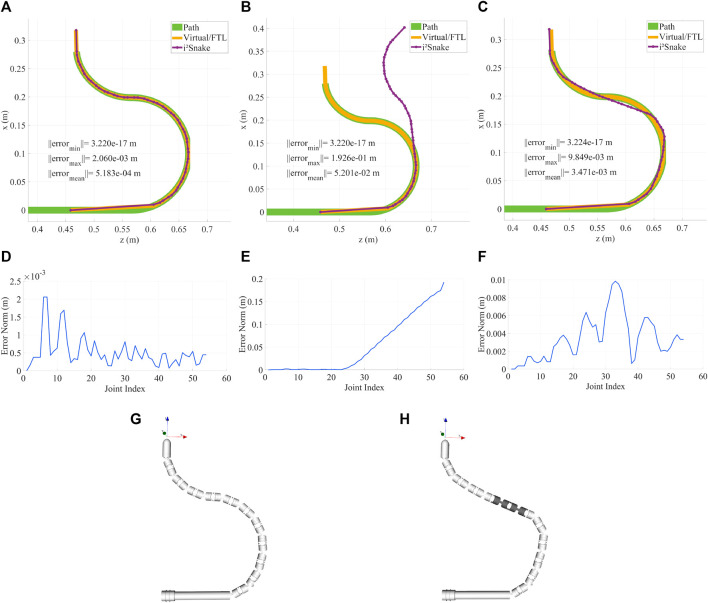
**(A,D)** Healthy *i* ^*2*^ *Snake* robot that can follow the path and corresponding error plot. **(B,E)** *i* ^*2*^ *Snake* robot with a faulty 4^*th*^ section and corresponding error plot, there is a significant difference between the real and desired robot position. **(C,F)** Same faulty 4^*th*^ section but with fault tolerance activated and corresponding error plot. The solver now compensates the faulty joints with the healthy ones to stay as close to the path as possible. **(G)** Rendering of the healthy *i* ^*2*^ *Snake*. **(H)** Rendering of the faulty snake with the faulty section highlighted.

The results of fault tolerance are shown in Figure 11. In this Figure, a) and g) depict a normal ‘healthy’ robot that can follow the trajectory with a maximum error of 2 mm. The detailed trajectory error is plotted in d) and shows that most links can be fitted with a sub-mm accuracy. b) Shows a faulty *i* ^*2*^ *Snake* with a damaged 4^th^ section with the faulty joints in a close to neutral position. The corresponding positioning error keeps increasing passed the faulty joints as shown in e). c) Shows the same faulty *i* ^*2*^ *Snake* with the fault tolerance feature activated.

It can be seen that the algorithm is able to find a solution to fit the faulty *i* ^*2*^ *Snake* as close as possible to the desired path which would not be possible with a simple approach such as stopping the actuation of the faulty tendon as shown in Figure 11 b). f) Shows the positioning error that increases around the faulty joints. An important matter to consider while performing fault detection is that, although a faulty joint can be detected, knowing the faulty joint angle is important to accurately perform compensation. In Figure 11, it was assumed that all the joints would stay almost straight as the hardware running inside of the *i* ^*2*^ *Snake* (instrument channels, Bowden cables, camera’s wires, sheath, *etc*.) would act as a spring keeping the joints almost straight as in traditional endoscopy.

An alternative would be to estimate the faulty joint angle by interpolating the angles between the adjacent healthy joints. This assumption was made only to show the potential of fault tolerance. Real-life applications would either require individual internal joint sensing, *e.g.*, optical sensing (Schmitz et al., 2017), FBG sensors (Liu et al., 2015) or external shape sensing with a sheath that can also sense external contacts (Wasylczyk et al., 2018). In any of these cases, MOVE would be able to perform the compensation as long as the faulty joint angles can be estimated.

### 3.9 Whole body solver performance

The performance of the solver was evaluated to assess its real-time capabilities. To evaluate the real-time performance, a trajectory was pre-recorded using the simulator. This trajectory recorded in the master ‘space’ is then used to generate a trajectory in the “slave” space that can be fed to the solver (solver trajectories are robot dependent as they depend on the amount of joints). The “slave” trajectory is then fed to the solver at varying frequencies (from 1Hz to 2 kHz) which means that the higher the frequency, the less time the solver has to converge to a solution. In the case of the extended *i* ^*2*^ *Snake*, the full-body Jacobian size is (165 × 54), and reaches (381 × 126) with *n* = 126 in the most complex robot architecture tested.

**FIGURE 12:**
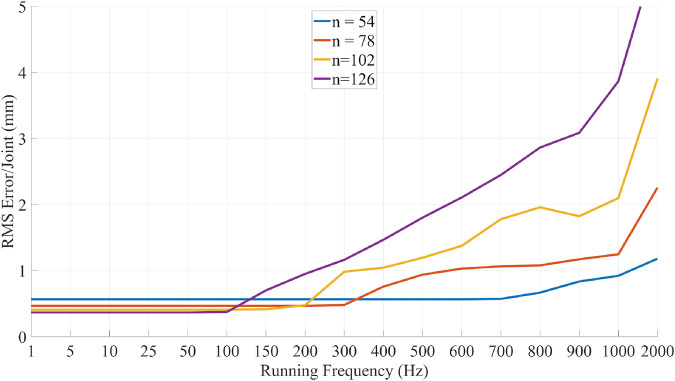
Performance evaluation of the proposed MOVE solver. A pre-recorded trajectory is fed to the solver at increasing frequency reducing the allowed time to converge to a good solution. The results show the averaged RMS error per joint. For the extended *i* ^*2*^ *Snake*(*n* = 54) the solver can run at frequencies of up to 1 kHz with sub-mm accuracy.

Figure 12 illustrates the corresponding results. The evaluation was performed using several *i* ^*2*^ *Snake*-like robots architecture with increasing complexity with ‘n’ being the amount of joint variables before mechanical coupling. The plots show the averaged RMS error between the link position and the path. The initial starting error is not zero as the *i* ^*2*^ *Snake* is not able to exactly follow the path. For the extended *i* ^*2*^ *Snake*, the error does not significantly change up to 700 Hz and can run at more than 1 kHz with sub-mm accuracy. The error of more complex robots is lower at low frequency as they have more joints and hence the average error per joint is decreased.

## 4 Real robot implementation

**FIGURE 13:**
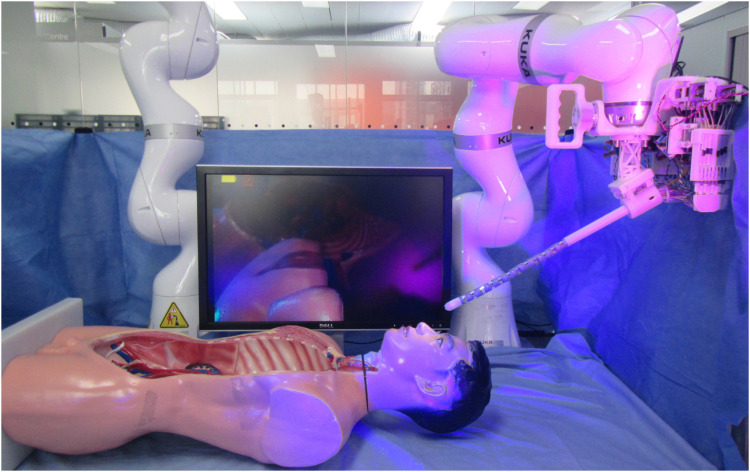
MOVE framework implementation on the real *i* ^*2*^ *Snake* robot. The system is composed of a KUKA robotic arm, the *i* ^*2*^ *Snake* and a head phantom. The patient would lie on the back or eventually on the side during the procedure.

**FIGURE 14:**
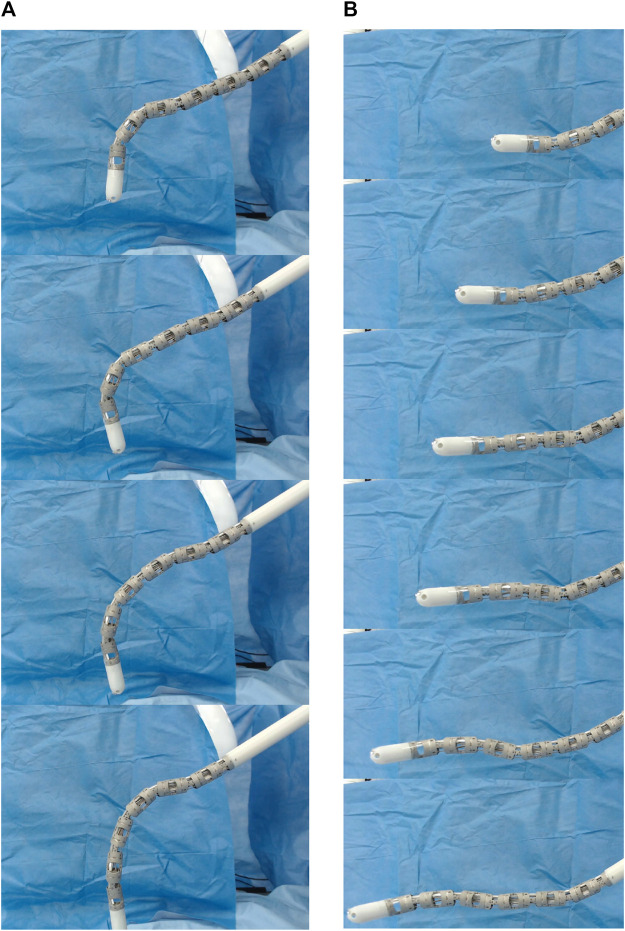
Time lapse of MOVE implemented on the *i* ^*2*^ *Snake* robot during a vertical motion **(A)** and a horizontal motion **(B)**. The insertion motion is provided by the Kuka robot.

The MOVE framework was implemented and tested on the real *i* ^*2*^ *Snake* robot presented in (13). The setup consists of an iiwa 7 robotic arm (KUKA, Germany) holding and providing the insertion motion to the *i* ^*2*^ *Snake* robot as shown in Figure 13. During a clinical application, the patients would lie on their back or on the side and the *i* ^*2*^ *Snake* would be inserted from the top of the head as depicted in Figure 14.

For the control, a laptop computer with an intel i7-8750H CPU and 16 Gb of RAM was used to run the MOVE framework and to teleoperate the navigation using keyboard input. The control is done as described in Section 2 using the following keyboard keys.

• ‘I’ uses the Kuka robot to do the insertion motion.
• ‘O’ uses the Kuka robot to do the retraction motion.
• ‘Up arrow’ moves the head of the *i* ^*2*^ *Snake* up.
• ‘Down arrow’ moves the head of the *i* ^*2*^ *Snake* down.
• ‘Left arrow’ moves the head of the *i* ^*2*^ *Snake* left.
• ‘Right arrow’ moves the head of the *i* ^*2*^ *Snake* right.
• ‘7’ moves the head of the snake counter-clockwise.
• ‘9’ moves the head of the snake clockwise.

The purpose of this hardware implementation is to validate on a real robot the MOVE concept. During clinical applications, the control would be done with a different master interface such as a joystick or a custom made master manipulator. Two types of motion were tested: vertical and horizontal insertion as shown in Figure 14 and in the attached video. It can be seen that the *i* ^*2*^ *Snake* performs the MOVE smoothly as validated in Section 3. As the *i* ^*2*^ *Snake* is inserted, the rest of the body follows the path taken by the head. No further characterizations were performed on the joint motion accuracy of the physical robot. The *i* ^*2*^ *Snake*’s joint motion characterization has been previously presented in (34) and further characterization is beyond the scope of this paper. The current *i* ^*2*^ *Snake* hardware is currently being further developed to improve the overall positioning accuracy.

## 5 Discussion

### 5.1 Parameter tuning

There are various parameters that can be tuned in the MOVE framework. The error tolerance function and parameters can be tuned to either match the size of the lumen or to define a soft limit, hence allowing to use the available space. This function was implemented to ensure a smooth transition between the forbidden and the allowed regions (rather than a ‘bang-bang’ type of control), but other types of functions could also be used. While in the allowed region, the other parameters such as tendon-stress reduction will induce joint motion, but once in the forbidden region, the solver will always aim to converge to the minimal error and hence override these factors.

Regarding the path sampling resolution, it is defined in the workspace of the robot. It determines the level of precision of the path taken by the robot. Hence, it is task dependent rather than robot dependent. If the resolution is 1 mm, additional control points will only be added if the robot motion is greater than 1 mm. For navigation through the oesophagus, a sub-mm resolution is sufficient clinically. In the case of lung, endovascular, or brain application, this resolution should be smaller.

### 5.2 Sensor integration

The fact that the *i* ^*2*^ *Snake* cannot always satisfy the desired path is an intrinsic design limitation which cannot be compensated by a simplistic FTL navigation. The *i* ^*2*^ *Snake* cannot intrinsically perform FTL motion since its joints can only move in one direction. The concept of MOVE is to allow the use of the surrounding space in a controlled way. In Figure 6A, we show the problem of pure FTL navigation with no controlled error. The resulting behaviour of the robot is unpredictable as it is dictated by the kinematics equations of the solver and different joint configurations will result in different behaviours. With the additional features of MOVE introduced such as error tolerance and weights, it is now possible to control the error between the robot and the path. This behaviour is much safer for surgical applications.

The use of sensors could further improve the safety by avoiding excessive pressure on the lumen. In practice, the surgeon must be aware of the limitations of the system (as in traditional surgery or robotic surgery; all instruments have limitations) but it should be handled by the robot and not the operator. The use of sensors could alert if an excessive pressure is detected. If the lumen is closing on each side, which is likely to happen during endoscopic application, the ideal approach would be to balance the pressure on each side to ensure a safe navigation. In all cases the system should have a maximum pressure allowed to avoid the tearing of the lumen.

During endoscopy, excessive forces against sharp bends such as in the colon can result in patient injury. In the context of MOVE, this risk is significantly reduced, as the robotic endoscope will actively follow the path without relying on anatomical structures as a guide. However, it is inevitable that the patient moves during the procedure, which could result in unexpected anatomical change and excessive contact force between the robot and the anatomy. This can be avoided if the robot is equipped with force or contact sensors. The sensor information can be used to alter the virtual robot fitting, so the new fitting point is away from the head’s path and in a contact free area.

**FIGURE 15:**
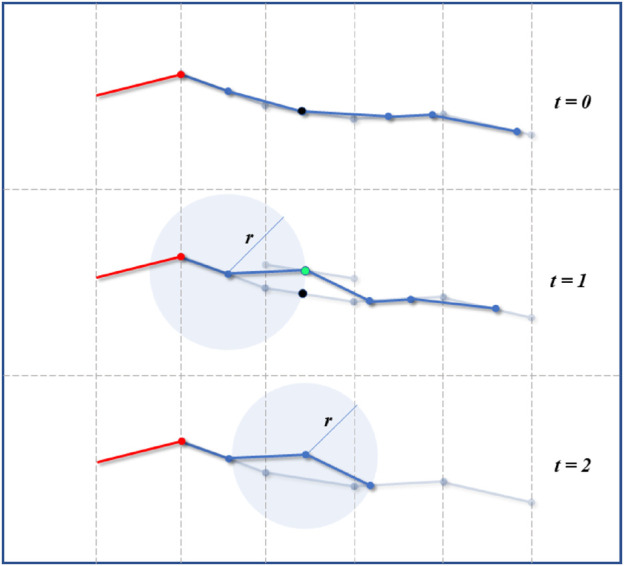
Virtual robot adjustment. In case of unexpected contact detected by force or contact sensors (black point at *t* = 0 and *t* = 1), the virtual fitting can be adjusted to find a contact-free point (green point) away from the path. Once the point adjusted, the fitting continues as normal.

This process could consist of translating the head path segment where a contact was detected, and to translate this segment opposite to the contact and normal to the contact direction as illustrated in Figure 15. The new fitting point would be located at the intersection between the translated path segment and the link’s sphere as in the normal fitting procedure. Once a safe point is found, the fitting could continue as normal.

## 6 Conclusion

In conclusion, this paper presents the MOVE framework for full-body control of snake-like surgical robots. The proposed framework is not system specific as it uses the standardized DH convention, so it can be applied on many different types of articulated snake-like robots.

The key contributions of the paper include a novel FTL-like navigation concept relying on the notion of extended full-body Jacobian matrix where the available surrounding space can be used, and finally a set of navigation algorithms to follow a desired path while ensuring reduced tendon stress and fault tolerance.

All the introduced new features were validated in simulation and the proposed solver’s performance was benchmarked for real-time performance. The results show that all the features of MOVE: path following, body weights, path weights, fault tolerance and conservative motion behave as expected. The solver can run in real-time on a standard computer at frequencies greater than 1 kHz. The MOVE framework was then successfully implemented on the real *i* ^*2*^ *Snake* robot as shown in the attached video.

Future work will focus on the sensing part of the MOVE framework to provide the system with real-time information on the available surrounding space and contact forces with the environment. Further work will also be dedicated to the *i* ^*2*^ *Snake* robot hardware and instruments to improve the overall precision and reliability of the system for clinical applications.

## Data Availability

The original contributions presented in the study are included in the article/Supplementary Material, further inquiries can be directed to the corresponding author.
